# PMCA Applications for Prion Detection in Peripheral Tissues of Patients with Variant Creutzfeldt-Jakob Disease

**DOI:** 10.3390/biom10030405

**Published:** 2020-03-05

**Authors:** Giorgio Giaccone, Fabio Moda

**Affiliations:** Fondazione IRCCS Istituto Neurologico Carlo Besta, Division of Neurology 5—Neuropathology, 20133 Milan, Italy; giorgio.giaccone@istituto-besta.it

**Keywords:** prion, PMCA, neurodegeneration, Creutzfeldt-Jakob disease, disease biomarker

## Abstract

Prion diseases are neurodegenerative and invariably fatal conditions that affect humans and animals. In particular, Creutzfeldt-Jakob disease (CJD) and bovine spongiform encephalopathy (BSE) are paradigmatic forms of human and animal prion diseases, respectively. Human exposure to BSE through contaminated food caused the appearance of the new variant form of CJD (vCJD). These diseases are caused by an abnormal prion protein named PrP^Sc^ (or prion), which accumulates in the brain and leads to the onset of the disease. Their definite diagnosis can be formulated only at post-mortem after biochemical and neuropathological identification of PrP^Sc^. Thanks to the advent of an innovative technique named protein misfolding cyclic amplification (PMCA), traces of PrP^Sc^, undetectable with the standard diagnostic techniques, were found in peripheral tissues of patients with vCJD, even at preclinical stages. The technology is currently being used in specialized laboratories and can be exploited for helping physicians in formulating an early and definite diagnosis of vCJD using peripheral tissues. However, this assay is currently unable to detect prions associated with the sporadic CJD (sCJD) forms, which are more frequent than vCJD. This review will focus on the most recent advances and applications of PMCA in the field of vCJD and other human prion disease diagnosis.

## 1. Introduction

Transmissible spongiform encephalopathies (TSEs) are a group of rare neurodegenerative conditions, also known as prion diseases, that affect both humans and animals [1]. Creutzfeldt-Jakob disease (CJD) is the most common form of human prion diseases that can have sporadic or genetic origins and can be acquired by infection [2]. In sporadic CJD (sCJD), the disease appears for unknown causes, affects 1–1.5 people per million annually, and accounts for at least 85 percent of cases [3,4]. Genetic CJD (gCJD) is caused by mutations in the prion protein gene (*PRNP*) and represents 10 to 15 percent of cases [5,6,7,8]. For instance, a point mutation at codon 178 of *PRNP* resulting in aspartic acid to asparagine substitution in coupling phase with methionine at position 129 (D178N/129M) leads to a disease named fatal familial insomnia (FFI) [9]. Finally, cases of iatrogenic CJD (iCJD) account for fewer than one percent of all known cases and have been reported in patients (1) subjected to corneal or dura mater grafts (whose donors died with CJD), (2) treated with human growth hormones (hGH) or gonadotropins derived from the pituitary gland of cadavers, and (3) subjected to brain surgery with contaminated electrodes (previously used in the brain of patients with CJD) [10,11,12,13,14,15,16,17]. The electrodes were sterilized with conventional procedures that were subsequently found to be inefficient to completely remove prion infection [18,19]. A serious health concern was raised after the demonstration that some forms of animal prion diseases could also be transmitted to humans [20]. For instance, the variant form of Creutzfeldt-Jakob disease (vCJD) is a zoonotic disorder that appeared in humans after the consumption of food products from cattle affected by bovine spongiform encephalopathy (BSE) [21]. BSE, also known as mad cow disease, was first discovered in 1986 in the United Kingdom and reached an epidemic peak in 1992 [20]. This resulted in stricter controls on cattle-derived food and many national CJD surveillance systems have been established [22,23,24,25,26,27]. Few cases of vCJD transmission between humans have been described in patients subjected to blood transfusion [28]. In particular, 4 among the 66 recipients who received blood products from asymptomatic vCJD donors were found to be infected [29]. Three of them developed the clinical signs of the disease and one remained asymptomatic [30]. For this reason, prions might be present in many other carriers who may never develop vCJD, but are capable of transmitting the disease. For this reason, specific preventive procedures are required to minimize the risk of secondary transmission of vCJD [27]. At present, no cases of sCJD transmission through blood transfusion have been described.

## 2. The Causative Agent of Prion Diseases

The causative agent of prion diseases is an abnormal form of the prion protein (PrP^C^), named PrP^Sc^ (or prion), which acquires pathological features and accumulates in the brain [31]. PrP^C^ is a glycophosphatidylinositol-anchored glycoprotein encoded by the *PRNP* gene located on chromosome 20 in humans [32,33]. Once synthesized, it undergoes three important post-translational modifications: (i) addition of the glycophosphatidylinositol (GPI) anchor, (ii) formation of a disulfide bond between cysteines at residues 179 and 214, and (iii) N-linked glycosylation at asparagine (Asn) residues at positions 181 and 197 [34]. For this reason, mature PrP^C^ exists in an unglycosylated, monoglycosylated, and diglycosylated form. The relative abundance of each form can be measured and quantified (by Western blot) to generate the glycoform profile [35]. The protein is rich in α-helices structures, soluble in detergents, and completely digested by proteinase K (PK) [31]. For reasons that are still not well understood, PrP^C^ can undergo structural rearrangements that lead to the formation of the pathological PrP^Sc^ [5]. Contrary to PrP^C^, PrP^Sc^ is characterized by the presence of a higher amount of β-sheet structures, is less soluble in detergents, and is partially resistant to PK-digestion [36,37]. After PK digestion, it is possible to detect a resistant core of 141 amino acids characterized by the presence of the diglycosylated, monoglycosylated, and unglycosylated PrP^Sc^ [38]. However, these bands migrate at a lower molecular weight compared with those associated with PrP^C^ [39,40]. Especially, the unglycosylated PrP^Sc^ band can migrate at either 21 kDa (N-terminus at glycine 82) or 19 kDa (N-terminus at serine 97), thus giving rise to type 1 and type 2 PrP, respectively [41].

Although being caused by the same protein, prion diseases are phenotypically heterogeneous [42]. Such differences are believed to be linked to different abnormal forms of PrP^Sc^, known as prion strains [43,44,45]. These strains can be characterized at a biochemical level by evaluating the pattern of PrP^Sc^ migration and the glycoform ratio (after PK digestion) [46]. Multiple factors, including the polymorphisms at amino acid 129 in the *PRNP* gene, are involved in human prion strains diversity [47,48,49]. For instance, the presence of either methionine (M) or valine (V) at codon 129 of the *PRNP* gene and PrP typing (1 or 2) gives rise to six different strains and clinicopathological phenotypes of sCJD: sCJD129MM1, sCJD129MM2, sCJD129MV1, sCJD129MV2, sCJD129VV1, and sCJD129VV2 [43]. sCJD strains are generally characterized by a predominance of the monoglycosylated PrP^Sc^ band [50]. By contrast, the vCJD strain shows a predominant diglycosylated band, while the unglycosylated one migrates at 19 kDa (type 2) [51]. For this reason, the PrP^Sc^ associated with vCJD has been classified as type 2B [52]. Regardless of the strain and through mechanisms that are still not well known, PrP^Sc^ can interact with PrP^C^ and force it to convert into new PrP^Sc^ [53]. Thus, at the expense of PrP^C^, PrP^Sc^ can spread in the central nervous system (CNS) while causing extensive neurodegeneration and ultimately leading to the death of the host [54]. By exploiting the capacity of PrP^Sc^ to convert PrP^C^ in vivo, Claudio Soto and collaborators have developed an assay named protein misfolding cyclic amplification (PMCA), which reproduces this phenomenon in vitro, in an accelerated manner [55]. This technique is currently being used in specialized laboratories for basic research on prion biology and diagnostic applications [56].

## 3. Protein Misfolding Cyclic Amplification (PMCA)

The protein misfolding cyclic amplification (PMCA) mimics in vitro the process of prion propagation, which occurs in vivo [55]. PMCA requires the incubation of prions with an excess of PrP^C^ in a test tube, which is placed in a dedicated sonicator and subjected to a process of cyclically repeated phases of sonication and incubation (referred to as *round of amplification*). Brains of healthy animals (collected from different transgenic mice or bank voles) are used as a source of PrP^C^. During the incubation phase, PrP^Sc^ forces PrP^C^ to change conformation and aggregate. The sonication fragments these aggregates into small species that act as seeds able to promote further PrP^C^ conversion. Each PMCA round has a variable duration (from 12 to 96 h). At the end of each round, an aliquot of the amplified sample can be diluted into a freshly prepared reaction substrate and subjected to an additional round of PMCA [57,58,59]. PMCA enables amplification of traces of PrP^Sc^ to a level that can be detected with the standard Western blot technique and, for this reason, has been introduced in the field of prion disease diagnosis [60]. However, in the case of human prions, this technology has an important limitation as it could only efficiently amplify the vCJD strain and, to a lesser extent, the FFI strain. Notably, amplified PrP^Sc^ maintained its biological and infectious properties, thus showing that the technology can faithfully replicate prions [61,62,63]. In particular, two different studies confirmed the ability of PMCA to generate vCJD prions that maintained the biological and infectious properties of the initial strain [64,65]. At present, no solid results indicate that PMCA can efficiently amplify other human prions, especially those associated with sCJD [66]. Certainly, these limitations depend on the experimental conditions used and increasing evidence suggests that their modifications could lead to a successful amplification of sCJD strains [66,67,68,69].

## 4. Prion Diseases Diagnosis: An Overview

The diagnostic protocol of CJD and prion diseases has deeply changed over the last decades. When TSEs were recognized as a disease entity following the intuition of C. Gajdusek (the first of the two researchers who received Nobel Prize for this topic, in 1976), the diagnosis of CJD could rely on the clinical signs and the course of the disease (usually rapidly progressive cognitive deterioration, ataxia, myoclonic jerks) and on peculiar changes at the electroencephalogram (EEG), consisting in pseudo periodic triphasic waves. Subsequent developments will show that this specific EEG change is found only in a subset of CJD patients.

Paralleling the paucity of diagnostic tools in vivo, the neuropathological diagnosis at this time was based on the demonstration of the histological hallmark triad of changes: astrogliosis, neuronal loss, and spongiosis. A major advance for the in vivo diagnosis of CJD was achieved in 1996 when Hsich and coworkers showed that the presence of proteins 130 and 131 belonging to the 14-3-3 family in the CSF correlates with a diagnosis of CJD, with high sensitivity and specificity [70]. The role of cerebrospinal fluid (CSF) was reinforced by successive studies documenting a marked increase of another neuronal protein, the microtubule-associated protein tau, in the CSF in the course of CJD [71]. Roughly at the same time, several studies appeared reporting bilateral and diffuse hyperintensities of the caudate nuclei and putamina at the magnetic resonance imaging (MRI) of the brain as a specific change of CJD [72]. From the neuropathological standpoint, one of the consequences of the prion hypothesis proposed by S. Prusiner (who received the second Nobel Prize for TSE in 1997) was the possibility to raise specific anti-PrP antibodies as tools for the detection of prions by immunohistochemistry (in fixed brain tissue) and by immunoblot (in frozen brain tissue), allowing the definite diagnosis of prion diseases based on the molecular alteration of PrP^Sc^, rather than merely on the morphological changes of cerebral tissue [73]. As previously described, this led to the recognition of two main types of PrP^Sc^ in the brain of these patients [43]. Thanks to these methodological advances, many patients with atypical forms of prion diseases could be definitely recognized and this substantially widened the spectrum of these disorders. Focusing on the genetic aspects, the identification of specific mutations in the *PRNP* gene causing rare genetic forms of prion diseases such as the Gerstmann-Sträussler-Scheinker disease and FFI heralded the identification of *PRNP* mutations in familial forms of CJD [74]. The definitive diagnosis of these diseases is generally based on DNA sequencing of blood samples. Genetic studies also contributed to the identification of molecular subtypes of CJD based on a combination of the type of pathological PrP and the polymorphism at codon 129 of PRNP [43]. 

In summary, the number of investigations for the in vivo diagnosis of prion diseases and in particular CJD has progressively and constantly increased in recent years, but among the tests and markers that can be carried out using tissues easily available in vivo, only PMCA, together with another cell-free amplification technique named real-time quaking induced conversion (RT-QuIC) [75], at difference from surrogate markers, roots its positivity on the direct detection of the basic molecular alteration, that is, the presence of PrP^Sc^ (even in minimal amounts), in peripheral tissues.

## 5. PMCA Applications in Human Prion Disease Diagnosis

As previously detailed, PrP^Sc^ is the only validated biomarker for prion diseases and definitive diagnosis relies on its postmortem detection in the brain collected at autopsy [31,76]. The process of PrP^C^-PrP^Sc^ conversion begins long before symptoms onset (even decades). When symptoms appear, huge PrP^Sc^ deposition has already occurred in the brain. Identification of PrP^Sc^ in peripheral tissues of patients with prion diseases has been hampered because of their minute amount, which could not be detected by conventional biochemical methods, even by bioassay in transgenic mice [77,78,79,80]. Thanks to the advent of the PMCA, traces of PrP^Sc^ were efficiently amplified and detected in peripheral tissues of vCJD and FFI patients, in some cases even at preclinical stages of the disease, thus representing a great revolution for the diagnosis of these disorders. However, vCJD and FFI account for only about 5% of all prion diseases. Recent evidence suggests that PMCA could potentially detect prion strains associated with the sporadic forms of CJD, which are more frequent than FFI and vCJD. If this were the case, PMCA would become a useful and indispensable tool for the diagnosis of all human prion diseases. Here, we summarize the diagnostic applications of PMCA reported so far.

### 5.1. PMCA Detected Prions in the Urine of Patients with vCJD

In 2014, we demonstrated for the first time that PMCA could detect prions in the urine of patients with vCJD [66]. Particularly, PrP^Sc^ was efficiently detected in 13 of 14 urine samples of vCJD patients, and in none of the 224 samples collected from patients with other neurologic diseases (including sCJD) and healthy controls. Our analysis was characterized by a sensitivity of 92.9% (95% confidence interval (CI), 66.1% to 99.8%) and a specificity of 100.0% (95% CI, 98.4% to 100.0%). We then performed a quantitative PMCA to estimate the PrP^Sc^ concentration, which was found to be at around 1×10^−16^ grams per milliliter of urine [81]. As a reaction substrate, we used the brains of mice genetically modified to express human prion protein with methionine homozygosity at *PRNP* codon 129 (TgHuMM). Urine samples were then prepared for PMCA analyses, which alternated cycles of incubation (29 min and 20 s) at 37/40 °C, followed by a 40 s pulse of sonication set at a potency of 270 to 280 Watt (Qsonica Q700 sonicator, Qsonica L.L.C., Newtown, Connecticut). Amplified PrP^Sc^ was characterized by a typical type 2B pattern (19 kDa, enriched in the diglycosylated form) [52,82]. To assess whether it also retained any infectious property, mice genetically modified to express human PrP with methionine homozygosity at PRNP codon 129 (Tg40) were intracerebrally inoculated with PrP^Sc^ either amplified from urine or contained in vCJD brain homogenates. Raw vCJD urine samples were injected as a control. Surprisingly, mice injected with urine amplified PrP^Sc^ and those injected with vCJD brain homogenate developed prion diseases that were characterized by similar clinical, biochemical, and neuropathological features. In contrast, none of the animals inoculated with raw vCJD urine developed prion pathology [64]. It is currently difficult to understand whether the urine of patients with sCJD contain PrP^Sc^ [83,84,85,86]. As a small quantity of PrP^Sc^ has been found in peripheral organs of these patients, it is likely that urine might also contain traces of prions [87,88]. To deepen this aspect, Tg40 mice were intracerebrally inoculated with urine samples from patients with sCJD. None of the animals developed prion disease and the study demonstrated that prion infectivity in urine is either absent or extremely low (below 0.38 infectious units/mL) [78,89]. At present, PMCA did not detect prion in the urine of patients with sCJD, however, this might be because of methodological limitations.

### 5.2. PMCA Detected Prions in the Blood of Patients with vCJD

In 2011, Edgeworth J. A. and coworkers developed the direct detection assay, which was the first assay able to detect vCJD prions in samples of whole blood. The technology was based on the use of a solid-state binding matrix to capture and concentrate PrP^Sc^ coupled to direct immunodetection of surface-bound material. A total of 190 whole blood samples, collected from patients with vCJD (*n* = 21), probable sCJD (*n* = 16), definite sCJD (*n* = 11), sporadic Alzheimer’s disease (*n* = 25), familial Alzheimer’s disease (*n* = 6), frontotemporal dementia (*n* = 4), other neurological conditions (*n* = 7), and normal controls (*n* = 100), were analyzed. The assay was able to identify vCJD samples with a sensitivity of 71.4% (15/21) and a specificity of 100% [90]. The results were confirmed in subsequent studies using large samples collected from healthy subjects and potentially cross-reactive populations [91,92]. These studies demonstrated for the first time that blood samples of vCJD patients contained infectious prions. In 2014, Lacroux C. and coworkers evaluated the possibility to exploit PMCA technology for detecting prions in the blood of cynomolgus macaques experimentally infected with vCJD, and subsequently in the blood of patients with vCJD. Their study demonstrated that blood collected from macaques intravenously inoculated with blood or brains obtained from vCJD-infected primates contained traces of prions detectable by PMCA. In particular, PrP^Sc^ was detected in the buffy coat of two macaques at 10- and 14-months post-inoculation, more than 960 days before the clinical onset of the disease. These results showed for the first time that prions circulated in the blood of macaques in the preclinical stages of the disease. Subsequently, they have shown the presence of PrP^Sc^ in white blood cells (WBCs)/buffy coat isolated from blood samples collected from three vCJD patients (out of four). As a reaction substrate, they used brain homogenate of mice genetically modified to express the ovine PrP with A_136_R_154_Q_171_ polymorphism. PMCA analyses alternated cycles of incubation (29 min and 30 s) at 39.5 °C, followed by a 30 s pulse of sonication set at a power of 70% (Misonix 4000x sonicator). They failed to detect prions in the buffy coat of one vCJD patient and PMCA was characterized by a limit of detection equivalent to 10^-7^ brain dilutions [93].

In 2016, two groups exploited PMCA for detecting prions in the blood of vCJD patients, sometimes even at pre-symptomatic stages of the disease. Concha-Marambio L. and colleagues optimized PMCA to analyze blood samples from 14 cases of vCJD patients (the same whose urine was also subjected to PMCA analyses, as described above [66]) and 153 subjects, including patients with sCJD, other neurodegenerative diseases, and healthy controls. In this work, before PMCA analyses, samples were subjected to high speed centrifugation for concentrating PrP^Sc^. After amplification, PrP^Sc^ was detected in all vCJD samples with a sensitivity and specificity of 100%. In particular, plasma and WBC fractions were found to carry PrP^Sc^ with an estimated concentration of about 5 × 10^−13^ grams per milliliter of blood [67]. As a reaction substrate, brains of TgHuMM mice were used. Then, 250 µL of blood samples was prepared for PMCA analysis (29 min and 30 s incubation at 37/40 °C followed by a 30 s pulse of sonication using an amplitude of 30—Qsonica Q700 sonicator). Likewise, Bougard D. and colleagues demonstrated that blood samples of patients with vCJD contain prions. However, compared with the work of Concha-Marambio, they used plasminogen-coated beads for capturing PrP^Sc^ present in 500 µL of plasma before PMCA analyses [94]. They analyzed blood samples collected from 18 vCJD patients at the clinical stage of the disease and 234 controls, including 67 patients with sCJD, other neurological conditions, and healthy subjects. PrP^Sc^ was detected in all vCJD samples with 100% sensitivity and specificity, regardless of the anticoagulant used to collect blood (ethylenediaminetetraacetic acid (EDTA) and citrate). Moreover, they found PrP^Sc^ in two non-leukodepleted plasma samples collected from donors before they developed vCJD, thus demonstrating that prions circulate in the blood even at preclinical stages of the disease (31 and 16 months before symptoms onset). Very recently, Concha-Marambio and colleagues showed that PMCA can detect prions in the blood of macaques peripherally infected with vCJD as early as two months post-inoculation (more than two years before the clinical onset of the disease), thus supporting the work of Lacroux published in 2014 [95]. As a reaction substrate, brains of TgHuMM mice were used. Then, 250 or 500 µL of blood was treated with sarkosyl to extract vCJD prions and subjected to PMCA analysis (29 min and 30 s incubation followed by a 30 s pulse of sonication using a Qsonica Q700 sonicator). At present, no cases of sCJD in subjects exposed to blood products have been reported. In 2017, Urwin P. and coworkers reported two cases of sCJD in patients with a history of extended treatment for clotting disorders. A link between the treatment and the onset of sCJD has not been established and these cases may reflect an accidental event [96]. However, in a study of Douet J. Y. and coworkers, inoculation of plasma samples collected from patients with sCJD in transgenic mice induced prion pathology, although with limited efficiency [77]. There is, therefore, an obvious need to optimize PMCA for the analyses of blood and blood products collected from patients with sCJD to further minimize the risks of potential disease transmission between humans.

### 5.3. PMCA Detected Prions in the Cerebrospinal Fluid of Patients with vCJD

In 2018, Barria M. and colleagues analyzed the cerebrospinal fluids (CSFs) collected from patients with vCJD (*n* = 15), sCJD MM1 (*n* = 6), and controls (*n* = 35) including patients with other neurodegenerative and neurological conditions [97]. CSF samples did not undergo any pre-treatment before PMCA analysis. As a reaction substrate, brains of mice homozygous for methionine at PRNP-codon 129 (HuMM) provided by Dr A Diack and Prof J Manson (Roslin Institute, University of Edinburgh) were used. CSF samples (7.5 µL) were added to PMCA substrates and subjected to cycles of incubation (29 min and 20 s) and sonication (20 s) using a Qsonica Q700 sonicator set at a potency of 280–300 Watt. Other than showing that PrP^Sc^ could be detected in the CSF of all vCJD patients included in the study with an estimated sensitivity of 100% (95% CI, 69.15%–100.00%) and a specificity of 100% (95% CI, 89.42%–100.00%), this group was able to analyze the CSF belonging to the first 129MV patients diagnosed with vCJD [98]. All vCJD cases diagnosed so far have occurred in patients homozygous for methionine at *PRNP* codon 129 [99]. During the same year, Boungard D. and coworkers analyzed more CSF samples collected from patients with vCJD (*n* = 41, including the MV patient), sCJD (*n* = 23), gCJD (*n* = 1), and controls (*n* = 33). This study showed a diagnostic sensitivity of 97.6% (95% CI, 87.1%–99.9%) and a specificity of 100% (95% CI, 93.7%–100%) [100]. They used the same experimental conditions reported in Section 5.2 (see also [94]). In particular, they performed PMCA experiments by alternating 15 min of incubation and 20 s of sonication. No successful amplification of prions in the CSF of patients with sCJD have yet been reported and this limits the PMCA technology to the diagnosis of vCJD.

### 5.4. PMCA Detected Prions in the Olfactory Mucosa of Patients with Fatal Familial Insomnia (FFI)

In 2017, Redaelli V. and coworkers adapted the PMCA technology for the detection of PrP^Sc^ in the olfactory mucosa (OM) of two patients with FFI [68]. Samples were collected in the late stage of the disease. As a control, 26 OM samples collected from patients with other neurodegenerative disorders, including Alzheimer’s disease, Parkinson’s disease, frontotemporal dementia, and healthy subjects, were used. None of them showed PrP^Sc^ amplification. Through quantitative PMCA, it was estimated that OM samples of FFI patients contained about 1 × 10^−14^ grams per milliliter of PrP^Sc^. As a reaction substrate, brains of bank vole carrying methionine at position 109 of PrP genotype (109 M) were used. Samples of OM (2 µL) were added to PMCA substrates and subjected to cycles of incubation (29 min and 30 s) and sonication (30 s) using a Qsonica Q700 sonicator set at a potency of 260–280 Watt. Surprisingly, using these experimental conditions, faint PrP^Sc^ amplification also occurred from the brain of patients with sCJD129MM1 and sCJD129MM2. This represents a preliminary proof of concept that sCJD can also be potentially amplified using PMCA [69]. At present, no studies have reported the possibility to detect PrP^Sc^ in OM collected from patients with vCJD by PMCA. This is likely because, in contrast to blood, urine, and CSF, such types of samples were not preventively collected from these patients. Similarly, there are no studies showing the ability of PMCA to amplify PrP^Sc^ in OM of patients with sCJD. However, as detailed below, traces of PrP^Sc^ could be efficiently detected in OM of sCJD patients using the real-time quaking induced conversion assay (RT-QuIC) [101]. Thus, the lack of prion detection in these tissues by PMCA seems to be closely related to methodological limitations that need to be overcome soon. Our group is currently working on PMCA optimization for the analysis of OM samples collected from sCJD patients and has gathered promising results that are currently under evaluation before any significant conclusion might be drawn. Besides the work of Redaelli et al. [68], no other studies on the capability of PMCA to detect prions in OM of patients with other genetic forms of prion diseases were reported. Similarly, crucial information about the infectivity of OM when challenged in mice is still lacking. It will be interesting to follow the growth of this field of research in the future.

## 6. Conclusions

The definitive diagnosis of prion diseases can be formulated only at postmortem by detection of PrP^Sc^ in the brain. Thanks to the advent of PMCA, traces of PrP^Sc^ have been mainly found in the urine, blood, and CSF of vCJD patients, even at preclinical stages of the disease. The discovery that vCJD could be transmitted between humans through blood transfusion has raised important concerns for public health, as there might be a self-sustaining secondary epidemic of the disease in the population [27]. For this reason, preventing the secondary transmission of the disease is an important priority, especially in the United Kingdom, and PMCA can be exploited either for screening blood and blood products to improve the safety of transfusion procedures or for identifying silent carriers of the disease that might contribute to the disease spreading. This is now even more meaningful after the description of the first vCJD case in a 129MV patient, thus suggesting that people who were thought to be less susceptible to develop the disease (those with MV and VV polymorphisms at *PRNP* 129) are instead susceptible, but likely with longer incubation periods compared with those of MM patients [98]. Finally, different prevalence studies (Appendix studies) revealed that prions can be found in appendices of U.K. people exposed to BSE who would never develop the disease, but are potentially able to transmit it [102,103]. Therefore, these findings have important implications for the management of blood products and for the handling of surgical instruments.

PMCA is currently being used to study another emerging form of animal prion diseases, named chronic wasting disease (CWD), which affects cervid species and might represent a new risk of zoonotic disease [104,105,106].

As sporadic CJD forms occur more frequently than vCJD in humans, it would be crucial to further optimize PMCA for detecting peripherally circulating PrP^Sc^ associated with sCJD. Having the possibility to detect and identify the individual PrP^Sc^ strain (i.e., PrP^Sc^ typing and glycoform ratio) from peripheral tissues of living sCJD patients would be extremely beneficial, eventually making the autoptic confirmatory tests no longer required for formulating a definitive diagnosis. Nowadays, only the RT-QuIC, which utilizes the same principle of PMCA, but slightly different technical procedures, enabled detection of PrP^Sc^ in CSF and OM of patients with sCJD with high efficiency [75,101,107,108]. In contrast to PMCA, which generally provides information about the biochemical features of the amplified PrP^Sc^, RT-QuIC indicates only whether the biological samples contain prions. Nevertheless, RT-QuIC is now a robust and validated tool employed for supporting the clinical diagnosis of prion diseases, especially sCJD [109]. Compelling evidence indicates that other neurodegenerative diseases, including Parkinson’s disease (PD) and Alzheimer’s disease (AD), which are more common than prion diseases, are caused by proteins (α-synuclein, amyloid-β, and tau) that possess properties similar to those of PrP^Sc^. Therefore, PMCA and RT-QuIC might be exploited for detecting peripheral disease-specific biomarkers associated with these more frequent disorders. In this regard, we and others have already shown that traces of aberrantly folded α-synuclein, amyloid-β, and tau can be detected in the cerebrospinal fluid and olfactory mucosa of patients with different neurodegenerative diseases [110,111,112,113,114,115,116,117,118,119]. However, these studies are in their very early phases of development and additional studies are required before these amplification technologies can be used as robust and reliable diagnostic tools.
